# Walkability, Overweight, and Obesity in Adults: A Systematic Review of Observational Studies

**DOI:** 10.3390/ijerph16173135

**Published:** 2019-08-28

**Authors:** João Paulo dos Anjos Souza Barbosa, Paulo Henrique Guerra, Crislaine de Oliveira Santos, Ana Paula de Oliveira Barbosa Nunes, Gavin Turrell, Alex Antonio Florindo

**Affiliations:** 1 Nutrition Department, Graduate Program in Public Health Nutrition, School of Public Health, University of São Paulo, Sao Paulo City 01246-904, Brazil; 2 Physical Activity Epidemiology Group, University of Sao Paulo, Sao Paulo City 03828-000, Brazil; 3 Federal University of Fronteira Sul, Chapecó Campus, Chapecó 89815-899, Brazil; 4 School of Arts, Sciences and Humanities, Graduate Program in Physical Activity Sciences, University of Sao Paulo, Sao Paulo City 03828-000, Brazil; 5 Centre for Urban Research, School of Global, Urban and Social Studies, RMIT University, Melbourne, VIC 3000, Australia

**Keywords:** walkability, environment, overweight, obesity, review

## Abstract

We conducted a systematic review to describe and summarize possible associations between the walkability index, overweight, and obesity. Systematic searches using seven electronic databases and reference lists were conducted to identify papers published until December 2017. Observational studies, describing associations using regression-based statistical methods, published in English and Portuguese, reporting markers of overweight and obesity, and involving adults (≥18 years) were included. Of the 2469 references initially retrieved, ten were used for the descriptive synthesis. Seven studies showed significant inverse associations between walkability and overweight and obesity, however, all were cross-sectional studies. High risk of bias scores were observed in “selection bias” and “withdrawals and dropouts”. All studies were published in high-income countries with sample sizes ranging among 75 to 649,513 participants. Weight and height as measures for determining BMI tended to be self-reported. Indicators of walkability, such as land-use mix, street connectivity and residential density were used as components of the indices. Based on this review, more studies should be conducted in low, middle, and middle-high income countries, using longitudinal designs that control neighborhood self-selection; other indicators of the neighborhood environment, such as food access, physical activity facilities, sidewalks, and safety and crime prevention should be considered.

## 1. Introduction

Overweight and obesity have risen dramatically over the last 40 years and they now constitute serious public health threats that are contributing to increasing rates of non-communicable disease such as type 2 diabetes and cardiovascular disease [1,2,3]. A recent publication gathering data from 239 prospective studies involving over 10 million individuals in four continents showed that the risk of mortality increases linearly with overweight (body mass index—BMI ≥ 25 kg/m^2^) and mainly obesity (BMI ≥ 30 kg/m^2^), regardless of age or sex [4]. A study of trends observed across 19 million participants shows a significant increase in BMI occurring over the last four decades, particularly in low- and middle-income countries. Between 1975 and 2014, the prevalence of age-standardized obesity increased 3.2% in 1975 to 10.8% in 2014 in men, and from 6.4 to 14.9% in women [5]. 

The major challenge faced relates to the fact that overweight and obesity are multi-factorial problems related mainly to behavioral variables, such as physical activity and diet [6], and these variables are related to health policies and living environments [7]. The built environment is defined as the physical form of neighborhoods, including patterns of land use, built and natural structures, and transportation systems [8]. The presence of an “obesogenic environment” hampers the adoption of healthy behaviors, such as maintaining a healthy diet, engaging in physical activity and limiting sedentary behavior [9,10,11,12,13]. Hence, obesogenic environments have emerged as the sum of influences that the surroundings, opportunities, or conditions of life have on promoting obesity in individuals or populations [9]. 

Walkability constitutes part of the built environment concept that can be operationalized when taking into account the setting and population under study and that appeared in the health field in the mid-2000s [14]. However, different built environment features have been used to calculate walkability scores, depending on the places in which studies have been conducted. In addition, some studies have shown that such results can vary according to the socioeconomic level of a given location and to neighborhood self-selection patterns [15,16,17,18]. Further, in upper middle income countries, other factors such as high population density levels and rates of violence, were related to rates of overweight and obesity [19]. Three previous systematic reviews stressed the importance of walkability in relation to obesity although they had a broader scope for both exposure and outcomes. The reviews examined more general issues, one of them synthesized current evidence on longitudinal relationships between a built environment and cardio-metabolic health outcomes among adults [20], the second was focused on obesogenic environments in high income countries [21], and the last review investigated which GIS-based measures of walkability (density, land-use mix, connectivity, and walkability indices) in urban and suburban neighborhoods were used and which of them were associated with active transportation and weight-related measures in adults [22].

Given these findings, a broad and up-to-date review of observational studies specifically on walkability, overweight, and obesity is crucial to better understand these relationships in different countries, how built environment components forming walkability are devised and assessed, how statistical models have been specified, and the main results that are found. Therefore, our main objective is to describe and summarize the evidence on associations between walkability, overweight, and obesity in adults. This review will also provide the basis for built environment interventions to help address the overweight and obesity crisis.

## 2. Methods 

The protocol of this review was registered on PROSPERO database (CRD42017071830). Its report is based on PRISMA checklist items [23]. 

The following inclusion criteria were adopted: (1) Observational studies (e.g., cross-sectional, cohort, time-series or case-control); (2) describing associations using regression-based statistical methods; (3) published in English and Portuguese; (4) reporting on overweight and obesity markers (5) that used walkability indices; and (6) using samples of adults (≥18 years). Articles not addressing the concept of walkability or that used separate indicators, that did not use markers of overweight or obesity, studies with children or adolescents, and qualitative and protocol studies were excluded. 

To retrieve potential references, seven electronic databases were used: Pubmed, Scielo, Lilacs, Web of Science, Scopus, Physical Education Index, and SportDiscus. Systematic searches were conducted in accordance with the strategy (Supplementary Materials S1: Search string) applied by Pubmed: ((((((((((((Neighborhood buffer[Text Word]) OR Neighborhood context[Text Word]) OR Walking locations[Text Word]) OR Space syntax[Text Word]) OR Street layout[Text Word]) OR Street design[Text Word]) OR Urban design[Text Word]) OR Urban form[Text Word]) OR Urban planning[Text Word]) OR Walkability[Text Word]) OR Walkable[Text Word])) AND ((((((Obesity[TextWord]) OR Overweight[Text Word]) OR Body Size[Text Word]) OR Body Weight[Text Word]) OR Body Mass Index[Text Word]) OR Adiposity[Text Word]). Using Lilacs and Scielo, searches were made in Portuguese using other terms such *“caminhabilidade”* and *“obesidade”, “sobrepeso”, “índice de massa corporal”*. Searches covered references available to December 2017. To avoid potential losses, manual searches through reference lists of the included studies were also performed.

Three researchers selected studies independently. Based on the systematic search results, titles and abstracts were screened, and then eligibility through a full text assessment was determined by all three researchers. Doubts and disagreements were resolved through a consensus meeting with a senior reviewer.

Data extraction involved the use of relevant information: Author (year), country/city/province/state (year of data collection), sampling, (n) sample, percentage of females, age (mean or range in years), and study type. Assessment of walkability variables: Geographic scale (e.g., census sectors, home addresses, post codes or geocoding buffers); geocoding of facilities; how walkability indicators were created and calculated. Assessments of overweight and obesity: How the markers were used (as categorical or dichotomous continuous variables); and the regression model used for analyses. Variables related to walkability, overweight, and obesity were extracted from included studies as significant or not significant associations at the 95% confidence interval or “*p*” values to better describe the odds ratio, beta coefficient, prevalence ratio, relative risks or other values with variables adjusted.

Risk of bias was evaluated with an adapted 12-item version, of the Effective Public Health Practice Project (EPHPP), proposed by Thomas et al. [24]. Other details of the assessment tool are given in Supplementary Materials S2. Original articles were assessed across seven methodological domains: (a) Selection bias (characteristics of the sample); (b) study design (information on the representativeness of the study, sampling methods); (c) confounders (control of relevant confounding factors of the analysis); (d) blinding (of assessors, outcomes and participants); (e) information on tools used to assess walkability and overweight/obesity (reports on previous validity and information, allowing for the reproducibility of the assessment of walkability and overweight/obesity); (f) withdrawals and dropouts (reported numbers and/or ratios and percentages of participants that completed the study); and (g) analysis (use of appropriate methods for analyses). The studies included were then classified by two previously trained researchers as presenting low, moderate or high levels of bias. 

## 3. Results 

**Figure 1:**
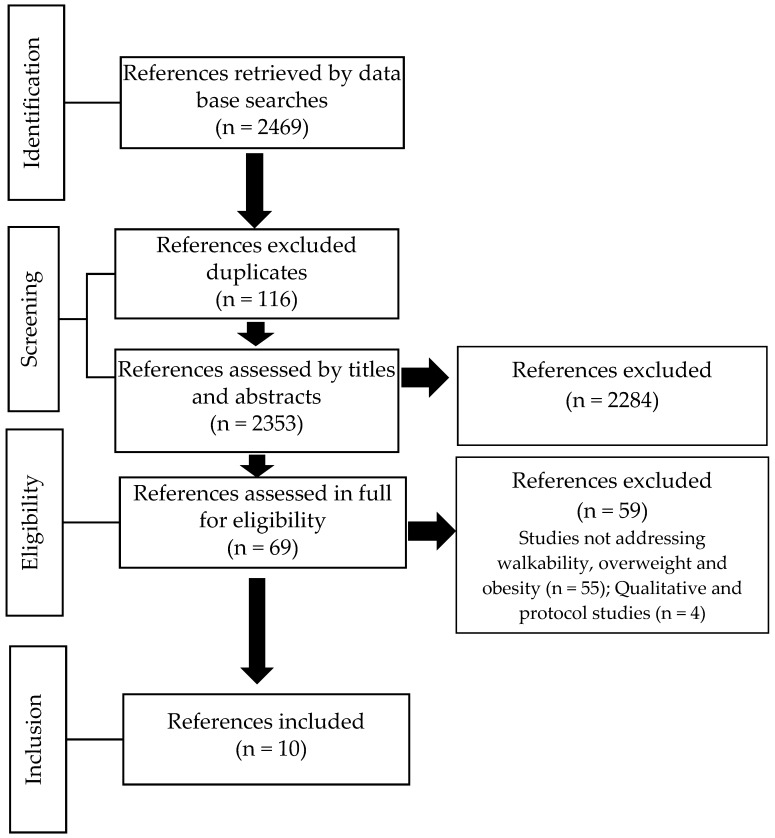
Systematic review flowchart.

Systematic searches retrieved 2469 potential articles. After the identification and removal of duplicate references (n = 116), 2353 references were screened by their titles and abstracts. Of these references, 69 were assessed from the full text. In view of the 59 rejected articles (for not addressing the main focuses of this review (n = 55) and for not reflecting qualitative and protocol studies (n = 4), 10 articles were used for the descriptive synthesis [15,25,26,27,28,29,30,31,32,33] (Figure 1). 

Sample sizes ranged from 75 [29] to 649,513 participants [30] (Table 1). Regarding available data on as a percentage of female participants (n = 7), females predominated in four studies [15,27,28,31], and only three studies involved a lower percentage of females than males [25,29,32]. The youngest participants were 18 years old and the oldest was 90 years of age [25,32]. All studies were conducted in high-income countries, particularly in the United States (n = 6) [15,27,29,30,32,33], Canada [25,26,28], and Australia [31] (Table 1).

Six studies employed a cross-sectional design (58.3%) (Table 1) [15,29,30,31,32,33], two employed a longitudinal design [25,27] one applied both cross-sectional and longitudinal methods (n = 1) [26] and one used time-series analysis methods [28]. 

Of the three studies employing a longitudinal design, none showed significant results [25,26,27]. Most of the studies that found an inverse association between walkability and overweight and obesity were cross-sectional (n = 6) [15,29,30,31,32,33]. The study that used time-series analysis presented significant results for the walkability index and a lower prevalence of obesity [28].

Regarding assessments used to the measure walkability (Table 2), most studies investigated main spatial information through neighborhood geocoding by geospatial sector of interest (e.g., census tracts, home addresses or post codes) (n = 7) [25,26,27,28,30,32,33]. Of these studies, two used home addresses [27,32]. One measured walkability attributes (e.g., population density, street connectivity, food, and physical activity resources) within three Euclidean kilometers using time-varying geographic information system (GIS) data [34] linked to participants’ geocoded home addresses [27]. Others used Mapping Analytics Inc., home addresses, walkability indicator population density levels, types of residences, median residence periods, and travel patterns [32]. 

Different buffer areas around residents’ homes were employed in two studies, e.g., a 1-km network buffer measured on the street network of each individual’s geocoded residence [15,29], and one study employed network buffers of 800 and 1600 m [31]. 

Variables used to measure walkability included residential and population density (n = 9; 91.6%), street connectivity (n = 8; 83.3%), and land-use mix (n = 5; 58.3%). Five studies defined walkability as the sum of the scores of main indicators with the index divided into quintiles [28] or quartiles [15] or expressed as a continuous score [30,32]. 

Scales and standard stadiometers (n = 3) [27,30,32] or self-reported measures (n = 7) [15,25,26,28,29,31,33] were used to measure weights and heights and to derive BMI scores to classify individuals as overweight (25–29.9 kg/m^2^) and/or obese (≥30 kg/m^2^). One study evaluated waist circumferences calculated from anthropometry measurements [27].

While various statistical analyses were employed, most studies used logistic regression analyses (n = 5) [15,27,30,31,33], while multi-level analyses were adopted in one study [25], one study used Poisson regression analysis [28] and another three studies used linear regression [26,29,32]. 

**Figure 2:**
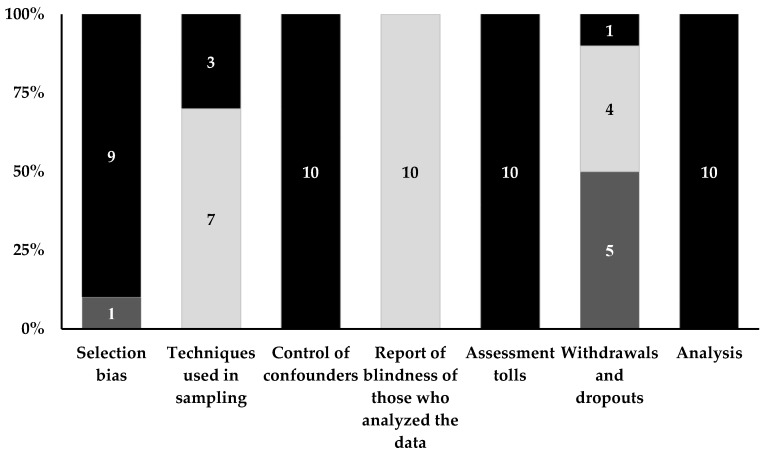
Risk of bias assessment of included articles (Black: low; Light grey: moderate; Dark grey: high). The numbers within the bars indicate the number of articles that were classified in the risk of bias classifications.

Eight studies adjusted and controlled variables employing different analysis models [25,26,27,28,29,30,31,32]. The main variables used for adjustment included sex, age, neighborhood socioeconomic status, ethnicity, physical activity, sedentary behavior, fruit and vegetable consumption, general health status, residential time, and the total number of household vehicles. Regarding the analyses by risk of bias, all included studies [15,25,26,27,28,29,30,31,32,33] were evaluated as low risk of bias in the domains “control of confounders”, “assessment tools”, and “statistical analysis” items. Moderate scores were observed in terms of “techniques used for sampling” (n = 7) [25,26,28,29,30,32,33], “report the blindness of the outcome assessor” (n = 10) [15,25,26,27,28,29,30,31,32,33], and “withdrawals and dropouts” (n = 4) [25,26,28,29]. High risk of bias scores were showed in “selection bias” (n = 1) [32] and “withdrawals and dropouts” (n = 5) [15,30,31,32,33] (Figure 2). 

Some of the previously reported results are noteworthy (Table 3), such as an increased prevalence of obesity observed in less walkable neighborhoods for quintiles of an 11-year period for adults in Canada [28]. One study focused on 2088 adults in Atlanta, USA shows that being poorer, older, Black, and from a larger household is related to greater odds of being obese [15]. Higher walkability scores are associated with lower rates of obesity (OR = 0.67; 95% CI 0.49–0.89) [15]. Lathey et al. (2009) found inverse associations for moderate (OR = 0.62; *p* < 0.05) and high (OR = 0.50; *p* < 0.001) walkability relative to low walkability indices for obese adult residents in Arizona, USA (June 2003 to June 2005) [30].

When stratifying by sex, walkability indicators present relations with obesity for both sexes and particularly for variables measuring destination diversity [33] like the land use diversity of a given neighborhood. 

When measuring “street design” in association with obesity, individuals living in highly compared with less walkable areas were less likely to be obese (1600 m OR: 0.84, 95% CI: 0.7 to 1; 800 m OR: 0.75, 95% CI: 0.62 to 0.9) [31]. 

Regarding other variables concerning walkability, only one study showed an inverse association between the proportion of inhabitants who walk to work and housing age (length of residence) to both men and women according to indicators of overweight and obesity. According to this study, the estimate of beta values was found to range from −6829 (*p* < 0.001) to −0.015 according to the BMI [33].

Three studies did not find associations between walkability, overweight, and obesity [25,26,27]. Two were longitudinal studies [25,27] while the other presented the results of cross-sectional and longitudinal analyses [26]. Despite not having found significant associations, it is important to note that these studies evaluated the changes in variables related to walkability as well as those of overweight and obesity. In addition, they referred to and discussed residential and neighborhood self-selection. 

## 4. Discussion

The aim of this study was to describe and summarize the evidence on associations between walkability and overweight and obesity. Seven out of ten included studies show significant inverse associations between walkability and overweight and obesity. Most studies show that less walkable neighborhoods are related to body weight outcomes in adult populations. Indicators measuring walkability index mainly include residential and population density, street connectivity and land-use mix. Some studies employed different buffer sizes around residents’ homes of 800 to 1600 m measured along street networks and network buffers. No studies involving longitudinal design had significant results.

Largest associations were found by cross-sectional studies, which generally support the incapacity to establish causality. In addition, all studies were conducted in high-income countries, which differ from low- and middle-income countries in their application of policies, higher levels of urbanization, broader employment opportunities, and greater availability and quality of public services (e.g., public transportation) [35,36].

The main indicators used to calculate the walkability index are commonly used in studies involving health areas and active transportation [37]. Other indicators include different aspects of urban design (e.g., block group-level measures), residence types and displacement patterns (e.g., traditional core, high density and non-auto commuting) [32]. Most walkability indices have been created by z scores of the different indicators and divided by the number of indicators as residential density, land-use mix and street connectivity. Some walkability indices were categorized in quintiles or quartiles or expressed as continuous scores and for this review we used the studies when the walkability index was calculated. Interestingly, from our synthesis, only one study used specific indicators of food environments and physical activity as indicators of the walkability index [27]. While these indicators may be related to land-use-mix variables, the use of such indicators helps strengthen the index given their known association with overweight and obesity [38,39,40,41]. 

Another important issue to discuss concerns the sizes of neighborhood buffers used. Buffers of 800 to 1600 m around residences were used in certain works [15,29,31]. Muller–Riemenschneider et al. investigated 800 and 1600 m buffers based “street design” in association with obesity levels and found individuals living in high compared with less walkable areas were less likely to be obese (1600 m OR: 0.84, 95% CI: 0.7 to 1; 800 m OR: 0.75, 95% CI: 0.62 to 0.9) [31]. Frank et al. used 1 km network buffers [15,29] around households based on street segments and found inverse associations between the walkability index and obesity. Using 1 km network buffers, one study of adults (n = 10,878) living in Atlanta, Georgia, USA from 2000 to 2002 shows that each quartile increase in land-use mix is associated with a 12.2% reduction in the likelihood of obesity across sex and race [42]. However, walkability was not used because the authors used main indicators separately and these were interpreted as measures of walkability.

Thus, studies examining the relationship between the walkability index and overweight and obesity appear to exhibit no consensus regarding buffer sizes. While some use distances that individuals can travel by walking for 10 or 15 minutes, which usually vary from 500 to 1600 m [43], other studies use buffers of 400 m to 8 km [8], denoting the difficulty of establishing a common parameter. The studies included in this review use GIS tools to study household participants and census tracts [25,26,27,28,30,32,33]. This poses a challenge to walkability research because some features such as the quality and aesthetics of spaces and facilities cannot be measured with secondary or remote data [33]. 

Based on assessments of bias risk, the results of the synthesis expose important methodological issues related to such studies, such as in the item “withdrawals and dropouts” for two studies [27,31]. This property can be considered a limitation mainly of longitudinal studies [27], as it is necessary to have a percentage of individuals remaining in the study at the final data collection period, and some longitudinal studies have been able to reassess at least 70% of the individuals with intervals between two or three years [17,44]. This issue should be considered in future studies. 

The assessment of certain predictors such as socioeconomic levels, demographic characteristics such as sex and other behavioral characteristics were used in the majority of studies examined in this review. Regarding socioeconomic levels, living in areas of higher socioeconomic levels may play a protective role against obesity [45], and this is interesting because socioeconomic status was used as an adjustment variable in different models and stratified the samples of some studies considered in this review [25,26,28]. Regarding sex, this predictor related to walkability is associated with overweight and obesity in both men and women and particularly among variables of destination diversity [33] (e.g., the land use diversity of a given neighborhood). 

Additionally, neighborhood-based changes in walkability can shape other behavioral characteristics, such as leisure, commuting, and physical activity measures [29,46]. Decisions regarding land-use and transport planning can influence, for instance, the safety of walking and cycling as modes of transportation and the convenience of recreational physical activity [35]. This is important given that urban planning that develops neighborhoods with better indicators for walkable neighborhoods promote walking as a mode of transport [35]. Thus, creating smart cities that facilitate physical activity as part of everyday activity can promote health and prevent overweight and obesity in the global population [35]. We found four studies [26,27,31,32] that put physical activity as adjustment variable in different models. It is important to note that the mediating effect of physical activity in such relationship involving walkability and obesity may not be found and some results still remain inconclusive, and this is confirmed in one recent systematic review [20].

Fruit and vegetable consumption is an important predictor with some studies revealing a relationship between diet and certain urban food environment land-use characteristics [34,47,48,49]. The availability [47] and variety [34,48] of healthy food is associated with diet, and supermarket density is related to higher levels of fruit and vegetable consumption [48] and to a reduced prevalence of obesity [50]. However, according to studies focused on the walkability index covered in this review, fruit and vegetable consumption significantly predicts BMI scores in a cross-sectional analysis model but not in a longitudinal analysis model [26]. One study using only longitudinal data and not using fruit and vegetable consumption as a predictor found that increased levels of obesity observed in lower-income neighborhoods are associated with issues of food accessibility [25]. Therefore, land use policies that protect and support access to healthful foods in urban areas are critical to mitigating differences in terms of access to local food.

### Strengths and Limitations

The strength of the present study was a broad review of observational studies specifically on walkability, overweight, and obesity, identifying which qualities contributed to walkability and how they measured or quantified these qualities and the association with overweight and obesity.

Most of the positive evidence has been obtained from cross-sectional studies that support the notion that certain neighborhood characteristics are related to low overweight and obesity prevalence [36]. Therefore, caution should be exercised when extrapolating these results due to neighborhood self-selection bias, as people who are not obese and who live a healthier lifestyle that prevents obesity may choose to live in neighborhoods with better living conditions [17,18]. To obtain stronger causal inferences, further longitudinal and quasi-experimental studies should be conducted in addition to natural experimental studies to further our understanding of how walkability at different urban scales affects risk of obesity [36]. Another limitation refers to the type of sampling of the included studies, four did not mention the type of sampling and two were convenience samples, the rest being studies with randomized samples. And because of this, it is possible that unmeasured confounders contributed to some findings.

Weight and height as measures for determining BMI scores were self-reported in the majority of the selected studies, collected through home-based and telephone surveys, and correctly cited reliable and previously validated information [25,28,29,33,51]. 

A broader variety of methodologies, including variables such as shade from street trees, the widths of sidewalks, safety and crime prevention, and others should also be employed and assessed in terms of walkability. Environmental determinants of obesity, including a healthy diet and certain food environments, such as supermarkets and restaurant chains, among others, are not addressed in the studies reviewed [38,39,40,41]. Few studies have explored walkability outside of the neighborhood setting (e.g., in areas surrounding workplaces). 

## 5. Conclusions

Most studies have found that less walkable neighborhoods are related to overweight and/or obesity in adult populations. Positive evidence has been obtained from cross-sectional studies and time-series studies, rather than longitudinal studies, and studies have been conducted in high-income countries. In addition, most studies have used a walkability index. Based on these results, the following recommendations can be made: 1) More studies should be conducted in low-income, middle-income, and middle-high-income countries; 2) more longitudinal studies (cohort and natural experiment) that control neighborhood self-selection need to be conducted; 3) other variables of the walkability index, such as food access, physical activity facilities, sidewalk access, and safety and crime prevention measured should be considered; and 4) better operationalizations of GIS evaluation variables (buffers sizes and census tracts) must be developed. Based on cross-sectional and time series studies, potential implications for clinical practice and policy-making can be reported, city planning and policy-related strategies aimed at improving the connectivity of the street network, mix of land uses and density of housing would enable the necessary supportive environments for health-related behaviors and prevention of chronic diseases. Understanding the factors that contribute to walkability can enable urban planners, designers and healthcare professionals to replicate better walkability conditions, providing more opportunities for active routes to help reduce overweight and obesity. 

## Figures and Tables

**Table 1:** Descriptive characteristics of the reviewed studies (n = 10).

Reference	Country/City or Province or State (Year of Data Collection)	Sampling	Sample Size (n)	%F	Age (Mean or Range in Years)	Study Type
Berry et al., (2010b) [26]	Canada/Alberta (2002/2008)	nd	1736 **	nd	<50/≥50	CS and L
Berry et al., (2010a) [25]	Canada/Alberta (2002/2008)	nd	500	47.8	18–90	L
Braun et al., (2016) [27]	USA (2000/2006)	R	1079	54.8	44.7	L
Creatore et al., (2016) [28]	Canada/Ontario (2001/2012)	nd	5500	51.3	30–64 (1)	TS
Frank et al., (2006) [29]	USA/Washington (1999)	R	75	44.5	20–65	CS
Frank et al., (2007) [15]	USA/Atlanta (2001–2002)	R	2088	50.6	40.9	CS
Hoehner et al., (2011) [32]	USA/Texas (1987–2005)	nd	16,543	30.3	18–90 (2)	CS
Lathey et al., (2009) [30]	USA/Arizona (2003–2005)	C	649,513	nd	35.5	CS
Muller-Riemenschneider et al., (2013) [31]	Australia/Perth (2003–2006)	R	5970	58.0	25–≥65 (3)	CS
Smith et al., (2008) [33]	USA/Utah (2007–2008)	C	453,927	nd	25–64	CS

Legends: ** longitudinal data for 572 participants and cross-sectional data for 1,164 participants; (1): Higher prevalence of people among 30–49 years old (66.7%); (2): Higher prevalence of people among 40–49 years old (39.1%); (3): Higher prevalence of people among 45–64 years old (40.7%); C: convenience; nd: not described; R: randomised; USA: United States of America; CS: Cross-sectional study; TS: Time-series study; L: Longitudinal study; %F: Percentage of females.

**Table 2:** Methodological characteristics of the descriptive studies.

Reference	Assessment of Walkability/Study Scale/Geocoding of Facilities	Walkability Indicators Evaluated	Walkability Index Calculated	Assessment of Overweight and Obesity/Markers of Overweight and Obesity	Regression Model Used for Analyses of Associations
Berry et al., (2010b) [26]	Geocoding of neighborhoods from census data	Residential density (density of dwellings per area in residential use), land-use mix (floor areas of the following five uses: residential, retail, office, education/institutional (including religious establishments) and entertainment) and street connectivity (density of true intersections in each neighborhood)	The walkability index was calculated based on Frank et al.’s formula not including the z-score for the retail floor area, and z-scores for walkability were then classified with quantiles (five classes of equal intervals divided into very high, high, moderate, low and very low walkability neighborhood groups).	Self-reported weight and height/BMI	Linear regression/standardized and unstandardized coefficient - β
Berry et al., (2010a) [25]	Geocoding of neighborhoods from census data	Residential density (density of dwellings per area in residential use), land use mix (floor areas of the following five uses: residential, retail, office, education/institutional (including religious establishments) and entertainment) and street connectivity (density of true intersections in each neighborhood)	The walkability index was calculated based on Frank et al.’s formula not including the z-score for the retail floor area, and z-scores for walkability were then classified using quantiles (five classes of equal intervals divided into very high, high, moderate, low and very low walkability neighborhood groups).	Self-reported weight and height/BMI	Ordinal regression model/standardized and unstandardized coefficient - β
Braun et al., (2016) [27]	Geocoding from home addresses	Population density, street connectivity and food and physical activity resources within three Euclidean kilometers of each respondent’s residential location.	The walkability index was determined from measures of population density, street connectivity, and food and physical activity resources measured from participants’ pre- and post-move residential locations.	Anthropometric measurements determined from exams/BMI and WC	Fixed effect regression models (logistic and linear)/odds ratio and standardized and unstandardized coefficient - β
Creatore et al., (2016) [28]	Geocoding of neighborhoods from the Canadian census	Population density (number of persons per square kilometer), residential density (number of occupied residential dwellings per square kilometer), walkable destinations (number of retail stores, services [e.g., libraries, banks, community centers], and schools a ten minute walk away) and street connectivity (number of intersections with at least 3 converging roads or pathways)	Neighborhood walkability derived from a validated index with standardized scores of 0 to 100 and with higher scores denoting more walkability. Neighborhoods were ranked and classified into quintiles from lowest (quintile 1) to highest (quintile 5) walkability.	Self-reported weight and height/BMI	Poisson Regression
Frank et al., (2006) [29]	Geocoding, 1 km network buffer	Net residential density (residential units divided by acres in residential use), street connectivity (intersections per square kilometre), land use mix (A/ln (N)) by entropy), and the retail floor area ratio (FAR) (the retail building floor area divided by the retail land area)	Sum of z-scores of land-use mix and net residential and intersection density	Self-reported weight and height/BMI	Linear regression/standardized and unstandardized coefficient - β
Frank et al., (2007) [15]	Geocoding, 1 km network buffer	Net residential density (residential units divided by acres in residential use), street connectivity (intersections per square kilometer), land use mix (A/ln (N)) by entropy), the retail floor area ratio (FAR) (the retail building floor area divided by the retail land area)	Sum of z-score land-use mix, net residential density and intersection density and divided into quartiles (lowest quartile, second quartile, third quartile and highest quartile)	Self-reported weight and height/BMI	Logistic regression/odds ratio and linear regression/standardized coefficient - β
Hoehner et al., (2011) [32]	Geocoding of neighborhoods by home address and residential block group	Traditional core (higher values corresponding to block groups with older homes and residents with shorter commute units), high density (higher values corresponding to block groups of higher populations and housing unit densities) and non-auto commuting (higher values corresponding to block-groups with a higher proportion of commute trips made by walking, bicycling, or public transport)	Block-group level measures of population density, housing type, median home age, and commuting patterns representing neighborhood walkability divided into different factors such as traditional core, high density and non-auto commuting. These factors were interpreted and analyzed separately	Weight and height/BMI	Fit regression model/standardized and unstandardized coefficient (β)
Lathey et al., (2009) [30]	Geocoding by census block group	Population density, land use, connectivity, locations for social interaction	The walkability index was calculated and divided into three groups: low (reference), average and high prevalence.	Weight and height/BMI	Multinomial logistic regression/odds ratio
Muller-Riemenschneider et al., (2013) [31]	Geocoding, 800 and 1600 m network buffers	Residential density (the ratio of residential dwellings to residential area in hectares), street connectivity (the ratio of three or more intersections to area in km2), land use mix (calculated with an entropy formula adapted from that developed by Frank et al. 2005 that considers the proportion of area covered by each land use type from the summed area for all land use types of interest divided by the number of land use classes)	The walkability index was calculated by summing the z-scores of each component	Self-reported weight and height/BMI	Logistic regression/odds ratio
Smith et al., (2008) [33]	Geocoding of neighborhoods by census block group - 2000 census for Salt Lake County, Utah	Population density, street connectivity, proportion of residents walking to work, the age of housing	Four D (density: population per square mile, design: intersections over 0.25 miles, diversity: proportion walking to work, and diversity: housing age) representing neighborhood walkability. These factors were interpreted and analyzed separately	Self-reported weight and height/BMI	Logistic regression/odds ratio and Linear regression/standardized coefficient - β

Legends: BMI: body mass index; WC: waist circumference.

**Table 3:** Synthesis of results.

Reference	Walkability Variables	Variable of Walkability (Score or Categorical)	Overweight and/or obese	Variable for Overweight and Obesity (Continuous/Categorical)	β-Values		Other-Values		OR-Values	95% CI	*p*-Value		Variables Adjusted	An Association (+) or No Association (ns) Found
	**Walkability-Index**													
Berry et al., (2010a) [25]	Lowest Walkability index	Categorical	BMI	Continuous	0.479						0.096		Age, sex, marital status, education, physical activity, fruit and vegetable consumption, neighborhood socioeconomic status	ns
	Low Walkability index				0.251						0.339			
	Moderate Walkability index				0.149						0.562			
	High Walkability index				0.3						0.232			
	Highest Walkability index (Reference)				-						-			
Creatore et al., (2016) [28]	Walkability index (Population density, residential density, walkable destinations (land use) and street connectivity) in quintiles absolute change adjusted values (prevalence)	Categorical	BMI	Categorical			Less walkable neighborhood (quintile 1) vs. most walkable neighborhood (quintile 5) = 43.3% vs. 53.5%	Adjusted prevalence					Age, sex, income and ethnicity	
								Quintile 1 (%) = 5.4		2.1%–8.8%	**0.002**			+
							Absolute difference = 10.2% (95% CI, 13.5% to 6.8%; *p* < **0.001**	Quintile 2 (%) = 6.7		2.3%–11.1%	**0.003**			
								Quintile 3 (%) = 9.2		6.2%–12.1%	**<0.001**			
								Quintile 4 (%) = 2.8		−1.4%–7.0%	0.20			
								Quintile 5 (%) = 2.1		−1.4%–5.5%	0.20			
Lathey et al., (2009) [30]	Walkability index (population density, land use, connectivity, locations for social activity) low (reference), medium and high	Categorical	BMI	Categorical					Low (reference)				Demographic and socioeconomic characteristics of a neighborhood	+
									Average = 0.62		**<0.05**			
									High = 0.50		**<0.001**			
Muller Riemenschneider et al., (2013) [31]	Less walkable vs. highly walkable neighborhoods (1600 m buffers)	Categorical	BMI (≥30 Kg/m2)	Categorical					Overall = 0.86	0.70–1.05	0.139		Age, sex, education level, household income, marital status, physical activity and sedentary behaviour	+
									Male = 0.82	0.59–1.14	0.229			
									Female = 0.88	068–1.14	0.336			
	Less walkable vs. highly walkable neighborhoods (800 m buffers)								Overall = 0.78	0.64–0.96	**0.018**			
									Male = 0.76	0.55–1.04	0.089			
									Female = 0.80	0.61–1.04	0.093			
Braun et al., (2016) [27]	Walkability index (population density, street connectivity, variables related to food and physical activity)	Score	BMI	Continuous	Fixed effects	Random–effects					Fixed effects	Random effects	Fixed effects adjusted for time (days between exams) and time-varying sociodemographic and health covariates (income, household size, marital status, employment status, smoking status, and health problems that interfere with physical activity) Random effects adjusted for time (days between exams), sociodemographic and health covariates (baseline age, sex, race/ethnicity, educational attainment, income, household size, marital status, employment status, smoking status, and health problems that interfere with physical activity), and reasons for moving to the current neighborhood (moved due to the built environment)	ns
					−0.022	−0.018					0.778	0.793		
			WC		−0.232	−0.26					0.391	0.283		
Hoehner et al., (2011) [32]	Walkability factors (traditional core, high density and non-auto commuting)	Score	BMI (women)	Continuous	Traditional core = −0.194						**<0.01**		Age and examination year and block group level percentage of non-Hispanic Blacks and Hispanics, percentage falling below the 200% poverty level, participation in outdoor physical activities (walking, jogging, or bicycling) and cardiorespiratory fitness	+
					High–density = −0.171						NS			
					Non-auto commuting = −0.028						NS			
			BMI (men)		Traditional–core = −0.210						**<0.05**			
					High–density = −0.158						**<0.001**			
					Non-auto commuting = −0.100						**<0.05**			
Berry et al., (2010b) [26]	Residential density, land use mix and connectivity	Score and categorical	BMI-(longitudinal)	Continuous	−0.068						0.116		Age, sex, marital status, education, physical activity, fruit and vegetable consumption, proximity to workplace, proximity to outdoor recreation amenities, quality of schools, quality of walking infrastructure, neighborhood socioeconomic status	ns
			BMI (cross- sectional)		−0.051						0.091			
Frank et al., (2006) [29]	Walkability index (residential density, street connectivity/land use, proportion of built area)	Score and categorical	BMI	Continuous	Unstandardized–coefficient	Standardized–coefficient							Sex, age, education, ethnicity, children under the age of 18 and household income	+
					−0.149	−0.113					**<0.001**			
Frank et al., (2007) [15]	Walkability index (residential density, street connectivity/land use, proportion of built area) in quartiles	Score and categorical	BMI	Categorical	–-				Lowest quartile (reference)	-	-		No variables adjusted	+
									Second quartile = 0.98	0.70–1.38	NS			
									Third quartile = 0.83	0.59–1.16	NS			
									Highest quartile = 0.67	0.49–0.89	**<0.05**			
	**Other variables**													
Smith et al., (2008) [33]	Density: population density	Score and categorical	BMI (women)	Categorical–and–continuous	0.000						0.663		No variables adjusted	+
	Design: street connectivity				0.000						0.981			
	Diversity: proportion of residents walking to work				−6.829						**<0.001**			
	Diversity: age of housing				−0.015						**<0.001**			
	Density: population density		BMI (men)		−0.001						0.336			
	Design: street connectivity				−0.002						0.092			
	Diversity: proportion of residents walking to work				−5.376						**<0.001**			
	Diversity: age of housing				−0.019						**<0.001**			

Legends: 95% CI: 95% confidence interval; BMI: body mass index; WC: waist circumference; OR: odds ratio; ns: no association found; bold values: significant *p*-Values.

## Supplementary Materials

The following are available online at https://www.mdpi.com/1660-4601/16/17/3135/s1, Supplementary Materials S1: Search string; Supplementary Materials S2: Explanation of 12-point EHPP tool used for risk of bias analysis.
