# Supplementary material for: Walkability, Overweight, and Obesity in Adults: A Systematic Review of Observational Studies
Source: Int J Environ Res Public Health. 2019 Aug 28;16(17):3135. doi: 10.3390/ijerph16173135 (PMC6747269; doi:10.3390/ijerph16173135)
Supplement: Supplementary file 1 [file ijerph-16-03135-s001.zip › Arquivo Comprimido 2 2/Supplementary file 2.docx]

Supplementary Materials S2 – Explanation of 12-point EPHPP tool used for risk of bias analysis.

**A) Selection Bias**

(Q1) Are the individuals selected to participate in the study likely to be representative of the target population?

Rating:

Low risk of bias: if the study sample is heterogeneous as to its health, that is, if it does not represent any specific clinical condition (e.g. diabetic samples).

High risk of bias: samples composed of people with common clinical conditions (eg pre-diabetic, diabetic).

**B) Study Design**

(Q1) Is there a description of the representativeness of the sample?

(Q2) Was the sampling method described?

(Q3) Was the method appropriate?

Rating:

Low risk of bias: Representative sample, composed by random method presented in the report of the article.

Moderate risk of bias: (i) non-representative sample, composed by random method informed in the article; (ii) representative sample, with sampling method not explained in the article (or in previous reference).

High risk of bias: (i) non-representative sample, arranged by convenience; (ii) non-representative sample, with sampling method not explained in the article (or in previous reference).

**C) Confounders**

(Q1) Relevant confounders were controlled (either in the design [e.g. stratification, matching] or analysis)?

Rating:

Low risk of bias: confounders controlled in analysis/ analyses

High risk of bias: analysed developed with no control for confounders

**D) Blinding**

(Q1) Was (were) the outcome assessor(s) aware of the intervention or exposure status of participants?

(Q2) Were the study participants aware of the research question?

Rating:

Low risk of bias: outcome assessor blinded in regard to expositions and also participants blinded in regard to main purpose of research.

Moderate risk of bias: (i) outcome assessor blinded in regard to expositions and participants do not blinded in regard to main purpose of research; (ii) no report for two questions; (iii) no report for Q1 and participants blinded in regard to main purpose of research;

High risk of bias: (i) outcome assessor do not blinded in regard to expositions; (ii) no report for Q1 and participants do not blinded in regard to main purpose of research.

**E) Assessment Tool**

(Q1) Previous validation report of the instrument used for the of walkability assessment

(Q2) Presentation of the instrument (e.g. as supplementary content) or report that allows the replication of the tool used to walkability assessment

Rating:

Low risk of bias: tool previously validated and its presentation or report that allows its replication.

Moderate risk of bias: (i) tool previously validated and no presentation or report that allows its replication; (ii) no report for two questions; (iii) tool do not validated and presentation or report that allows its replication.

High risk of bias: tool do not validated and no presentation or report that allows its replication.

**F) Withdrawals and Dropouts**

(Q1) Were withdrawals and dropouts reported in terms of numbers and/or reasons per group?

(Q2) Indicate the percentage of participants completing the study, considering the difference between those who signed the consent form and the number analysed.

Rating:

Low risk of bias: withdrawals and dropouts reported and analysis involving ≥80% of initial sample (those who signed the consent form).

Moderate risk of bias: withdrawals and dropouts reported and analysis involving 60–79% of initial sample (those who signed the consent form).

High risk of bias: (i) no report for Q1 and Q2; (ii) withdrawals and dropouts reported and analysis involving ≤59% of initial sample (those who signed the consent form).

**G) Analyses**

(Q1) Are the statistical methods appropriate for the study design? (S= 1; ND= 0; DI= -1)

Rating:

Low risk of bias: Yes

High risk of bias: No
