# Supplementary material for: Walkability, Overweight, and Obesity in Adults: A Systematic Review of Observational Studies
Source: Int J Environ Res Public Health. 2019 Aug 28;16(17):3135. doi: 10.3390/ijerph16173135 (PMC6747269; doi:10.3390/ijerph16173135)
Supplement: Supplementary file 1 [file ijerph-16-03135-s001.zip › Arquivo Comprimido 2 2/Supplementary file 1.docx]

Supplementary Material S1 – Search string

((((((((((((Neighborhood buffer[Text Word]) OR Neighborhood context[Text Word]) OR Walking locations[Text Word]) OR Space syntax[Text Word]) OR Street layout[Text Word]) OR Street design[Text Word]) OR Urban design[Text Word]) OR Urban form[Text Word]) OR Urban planning[Text Word]) OR Walkability[Text Word]) OR Walkable[Text Word])) AND ((((((Obesity[TextWord]) OR Overweight[Text Word]) OR Body Size[Text Word]) OR Body Weight[Text Word]) OR Body Mass Index[Text Word]) OR Adiposity[Text Word]).
